# Challenges and opportunities in patients with adult congenital heart disease, a narrative review

**DOI:** 10.3389/fcvm.2024.1366572

**Published:** 2024-05-30

**Authors:** Matteo Fabbri, Anurag Sahu

**Affiliations:** ^1^ Department of Cardiovascular Disease, Inova Heart and Vascular Institute, Falls Church, VA, United States; ^2^ Department of Cardiovascular Imaging, NIH/NHLBI Cardiovascular Imaging Lab, Bethesda, MD, United States; ^3^ Department of Cardiovascular Disease, University of Virginia School of Medicine, Charlottesville, VA, United States

**Keywords:** adult congenital heart disease (ACHD), pregnancy, heart failure, congenital heart abnormality, transition of care to adult facility

## Abstract

Adult congenital heart disease Pregnancy Transition of care Challenges heart failure.

## Introduction

Adult congenital heart disease (ACHD) is a field undergoing substantial growth due to the increasing patient population. As neonatal surgical techniques have evolved and medical therapies improved, the prevalence of adults with congenital heart conditions (CHD) has surpassed the number of children with CHD (1). Additionally, the spectrum of ACHD patients is heterogenous and represents varied anatomical lesions from isolated pulmonary valve disease to single ventricle physiology (2, 3).

This paper is designed as a targeted outline for healthcare providers into the field of ACHD, offering a focused overview.

## Appropriate ACHD care

ACHD patients faces multiple challenges during their lifetime. Among the first challenges is the transition of care from pediatric cardiovascular care to adult care. In fact, many patients are lost to care at the time of transfer of care or do not participate in a transfer program (4, 5). As few as 15% of ACHD patients may transfer to an ACHD center (5). There are several factors that account for this. ACHD patients typically have been under the care of pediatric cardiologists and other specialists since birth. Transitioning to adult care often requires leaving behind a familiar and supportive healthcare team, which can lead patients to feeling lost (6, 7). Moreover, there is a lack of awareness for the need of lifelong care among many ACHD patients and/or families (8, 9). Finding ACHD specialists can also be challenging as there is a shortage of such specialists particularly outside of major urban settings (10). In the United States, ACHD patients may also encounter difficulties with insurance coverage as they age out of pediatric coverage (7, 11, 12).

To address the drop-off in care, healthcare systems are increasingly recognizing the need for dedicated ACHD and transition programs (13). These programs aim to provide comprehensive care, education, and support to ACHD patients during the transition from pediatric to adult care, as well as throughout adulthood. They emphasize the importance of lifelong care, early education about the condition, and ongoing monitoring to prevent complications and improve the overall health and quality of life for ACHD patients.

**Figure 1:**
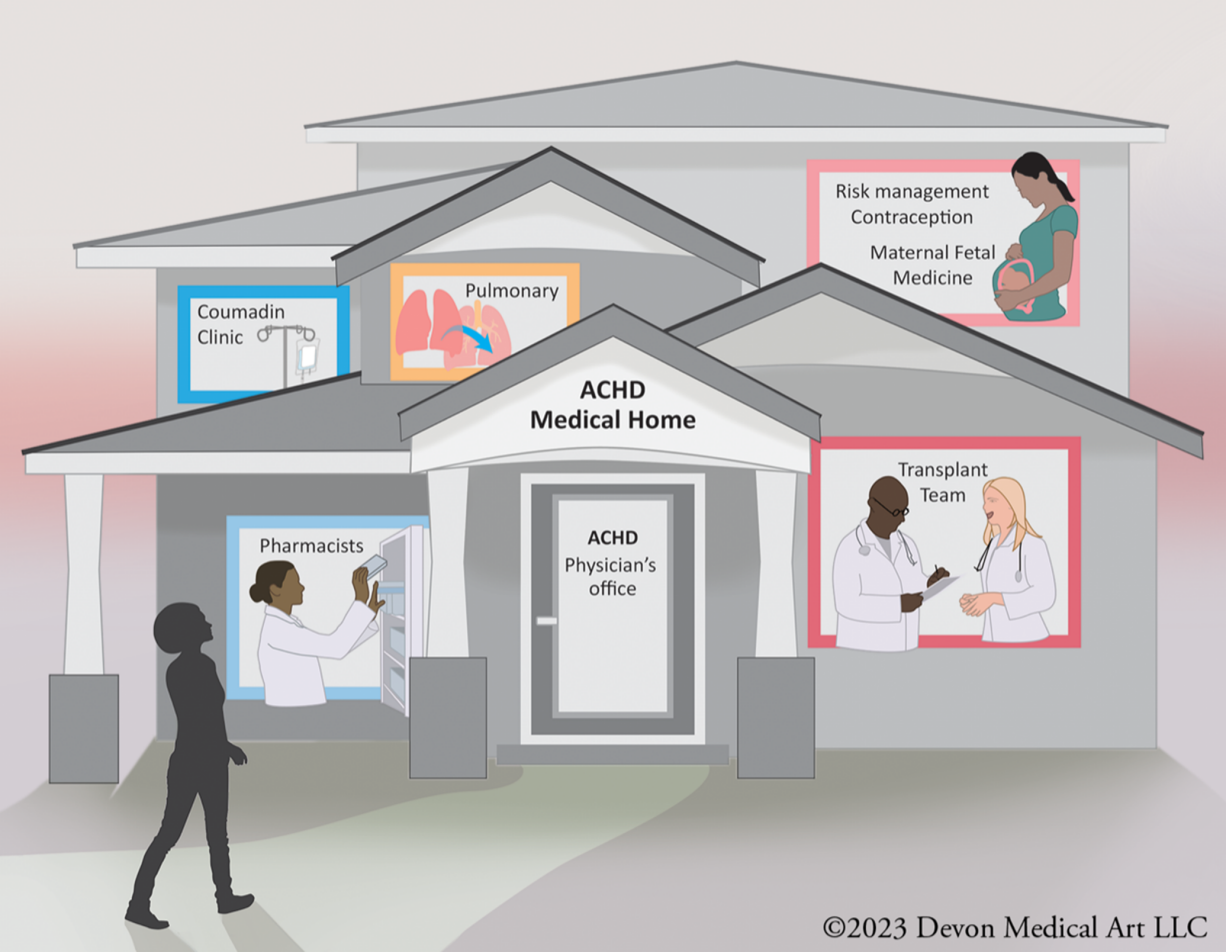
A multidisciplinary adult congenital heart disease (ACHD) team typically includes cardiologists, surgeons, nurses, and other healthcare professionals who specialize in managing complex cardiac conditions in adults with congenital heart defects. This team collaborates to provide comprehensive care, offering expertise in diagnostics, medical and surgical interventions, as well as emotional and psychological support for ACHD patients.

When these patients establish care in such a specialized center, they are usually managed by a multidisciplinary team of cardiovascular specialists. A comprehensive ACHD center is a specialized healthcare facility dedicated to providing comprehensive care to adults with CHD. These centers are designed to meet the unique medical, psychological, and social needs of ACHD patients throughout their lives. ACHD centers have a team of healthcare professionals with expertise in ACHD, including cardiologists, cardiac surgeons, nurse practitioners, social workers, psychologists, and other specialists. This multidisciplinary team collaborates to provide holistic care (Figure 1).

Perhaps a unique aspect of a comprehensive ACHD center that can be overlooked relates to genetic counseling services. While in the current era genetic counseling and evaluation is commonplace in pediatric cardiology, the current generation of ACHD patients may not have been evaluated by genetics at time of diagnosis. As such, patients may not recognize their own genetic syndrome or the recurrence risk in their children. In the ACHD clinic, a genetic counselor can initiate the appropriate screening and refer patients to a geneticist as needed. Additionally, they may suggest other subspecialty referrals based on medical history (14).

As mentioned previously, transition programs built as part of these comprehensive ACHD programs are crucial. While well-structured transition from pediatric care to ACHD care has been identified as a critical need in caring for congenital heart patients, the inability to appropriately transition patients can lead to harm such as inappropriate medical interventions or inappropriate care with consequently resultant heightened financial and emotional distress (15).

Individuals with severe congenital heart disease exhibit a higher likelihood of transitioning to adult congenital heart disease care compared to those with non-severe conditions. And even amongst those with severe disease, transfer rates to ACHD care have been reported be just below 20% (5).

Ideally, a transition program consists of a healthcare team including nurses, social workers, care coordinators and both pediatric and adult physician champions (7).

The transition process should begin early and at multiple visits so that appropriate anticipatory guidance can be provided in regard to possible future procedures and reproductive health for teenagers and adults. Additionally, appropriate counseling for behavioral health, exercise, and career choices should also be discussed. This also helps educate so that both family and the patient recognize the lifelong needs of ACHD patients, hence the need to maintain contact with the healthcare system.

Additionally, ACHD care also necessarily incorporates traditional adult cardiovascular care. Congenital heart survivorship is now the norm, and as such ACHD patients are also at risk for traditional cardiovascular events. Furthermore, the high prevalence of lipid disorders in patients with ACHD “as high as 69%” strongly supports the recommendation for regular screening for cardiovascular risk factors, particularly dyslipidemia, as part of routine ACHD care (16).

## ACHD surgical considerations

Surgery for ACHD presents a unique set of challenges compared to surgery for acquired heart conditions. ACHD patients often have complex and unique cardiac anatomy, therefore ACHD surgeons must have a thorough understanding of the patient's specific anatomy and be prepared to adapt surgical techniques accordingly.

Most surgeries in ACHD patients include valve repair or replacement as well septal defect repair. The European Congenital Heart Surgeons Association Database collected a total of 20,602 ACHD patients who underwent cardiac surgery, between January 1997 and December 2017. The most common procedural groups included septal defects repair (*n* = 5,740, 28%), right-heart lesions repair (*n* = 5,542, 27%) and left-heart lesions repair (*n* = 4,566, 22%); additionally, almost one-third of the procedures were re-operations (*n* = 5,509, 27%) (17).

Other studies have suggested that more than half of all operations performed on ACHD require repeat sternotomy (18). This can be explained by the evolving spectrum of ACHD patients. In its original form many ACHD patients consisted of patients requiring primary defect repair, while they now largely encompass patients who underwent primary repair as children and require treatment of residual defects or sequelae of the initial pathology or previous treatment. In fact, primary correction of the heart defect accounts for less than 25% of all operations in ACHD (18).

Assessing surgical risk in ACHD patients requires consideration of multiple factors. Foremost, some ACHD surgeries may not provide a permanent solution, particularly valvular surgery. Many patients require multiple interventions throughout their lives. ACHD physicians and surgeons must consider the long-term implications and potential for future reinterventions when planning ACHD surgery. Woman of childbearing age, as an example, may elect tissue aortic valve or Ross procedure over a mechanical valve to avoid the risks of coumadin during a future pregnancy. However, this is likely to lead to further cardiovascular procedures in such as transcatheter aortic valve replacement (TAVR), transcatheter pulmonary valve replacement (TPVR), or re-do sternotomy.

There has also been a shift in overall CHD-related mortality from childhood to adulthood. Specifically, more than half of all CHD-related mortality occurs in adulthood (19), while in-hospital mortality following congenital heart surgery in adults is also likely higher among adults (20). Up to 10% of ACHD surgical patients appear to suffer post-operative complication (18, 21). Early mortality following cardiac surgery is as high as 3.6% with single ventricle physiology with the number of sternotomies being an independent major predictor (21).

Fortunately, the majority of ACHD surgical interventions are elective and can undergo careful planning and risk assessment. Traditional calculators of surgical risk such as the European System for Cardiac Risk Evaluation (EuroScore II) exclude the ACHD population (22). ACHD risk score calculators such as the perioperative ACHD score calculator (PEACH Score) have been developed to specifically assess risk in patients undergoing ACHD surgery (23). A PEACH score of 0 predicts an in-hospital mortality of 0.2%, 1–2 predicted a mortality risk of 3.6%, and ≥3 (17.2%). Each risk factor is assigned one point: NYHA class 3 or 4, urgent surgery, active endocarditis, ≥2 prior sternotomies, Adult Congenital Heart Surgery Score >1.5, Hg <10 gm/dl or >20 gm/dl, and evidence of CKD with estimated GFR < 60 ml/min. Referenced in this algorithm is the Adult Congenital Heart Surgery Mortality Scoring system (24) which assigns cardiac risk based upon type of operation. Those scoring above 1.5 are considered some of the highest risk in the ACHD population. These include Fontan revision surgery, heart transplant, and lung transplantation. Interestingly, coronary artery bypass grafting (CABG) in the ACHD population was illustrated to be high risk. Although elective CABG is generally considered low risk in individuals who are otherwise healthy, when performed in conjunction with the complexities of previously corrected CHD, it seems to transition into a high-risk surgical procedure. Surgeons performing multiple grafts may face challenges accessing and grafting coronary arteries due to variations in heart anatomy. Additionally, those undergoing CABG often have ahead a prior sternotomy in addition to having other medical problems such as hypertension, diabetes, or kidney disease which can increase the risk of complications during surgery.

While these scoring models provide valuable guidance for predicting the risk of surgery in ACHD, they are not exhaustive. Various patient-specific factors also contribute significantly. Factors such as the presence of lung disease, obesity, pulmonary hypertension, systemic right or left ventricle, the necessity for multiple valve replacements, age, and liver disease are just a few examples of risk factors that influence the assessment of surgical risk in ACHD patients. Consequently, it is essential to take a comprehensive approach that considers the patient's unique anatomy, individual risk factors, and the potential benefits of the surgery when evaluating ACHD patients for cardiac surgery.

## Interventional considerations

One of the most significant advancements in ACHD care has been the proliferation of minimally invasive procedures. These procedures often involve catheter-based interventions. They offer several benefits including reduced surgical risks, shorter hospital stays, and faster recovery times. The development of percutaneous implantable heart valves has been uniquely suited for the treatment of ACHD patients. In fact, the first TAVR ever performed was done on a patient with CHD (25). As TAVR technology initially evolved for non-congenital patients, it has also result in significant growth in the field of CHD interventions.

ACHD patients such as those with conditions like tetralogy of Fallot or pulmonary atresia, often require pulmonary valve replacement in adulthood. The emergence of TPVR has provided a viable alternative to re-do sternotomy for such patients. TPVR is primarily employed in cases involving bioprosthetic pulmonary valve dysfunction and failure of the right ventricle to pulmonary artery conduit (26).

In the United States, the two most commonly used transcatheter pulmonary valves are the Melody™ Transcatheter Pulmonary valve by Medtronic Inc (27). and the Edwards Sapien by Edwards Lifesciences (28). However, this initial transcatheter pulmonary valve technology had limitations, particularly in accommodating patients with larger, compliant, and irregularly shaped right ventricular outflow tracts (RVOT). This led to the development of both the Harmony™ (29) and the Alterra Adaptive PrestentTM (30). These innovations find applications in the management of pediatric and adult patients experiencing severe pulmonary regurgitation, as diagnosed by echocardiography and/or with a pulmonary regurgitant fraction equal to or exceeding 30% by cardiac magnetic resonance imaging. These advanced technologies are suitable for individuals who have either a native or surgically repaired right ventricular outflow tract and have clinical indications for surgical pulmonary valve replacement.

Despite these advances, nonsurgical options for patients with a severely enlarged and often native or patched RVOT remain limited. Approximately half of the patients screened are ineligible for these devices because the length of the landing zone is either too short or wide (31–33). As such, novel therapies are often needed in these cases as well as others.

The evolution of transcatheter technology, not only in valve interventions but across the entire spectrum of CHD treatment, is a natural outcome of the collaborative efforts of multidisciplinary teams. In the multidisciplinary team model, each member brings their specialized expertise to the forefront. ACHD cardiologists contribute valuable insights into medical management and diagnosis, structural interventionalists offer recommendations on cath-lab therapies, and cardiac surgeons provide their wealth of knowledge on surgical options. Additionally, interventional radiologists or vascular surgeons may provide specific instruments or techniques that can be utilized in ACHD structural intervention but are not typically considered in the realm of cardiac structural interventionalist (34). Imaging specialists also play a critical role in reviewing relevant data with the team, ensuring that the diagnostic foundation is robust, while anesthesiologists prioritize the patient's well-being throughout the entire interventional/surgical process. This heart team approach, which has recently been well-established in the realm of structural interventions (35, 36), arguably finds its origins in the field of ACHD. ACHD care has long recognized the importance of multidisciplinary teams, dating back to the inception of comprehensive ACHD center models (26).

ACHD structural intervention includes therapy far beyond just pulmonary valve replacement. Structural intervention for baffle leak closures, ASD/VSD closures, coarctation of the aorta, and patent ductus closures are common. These procedures are tailored to the individual patient's anatomy and condition, with the goal of achieving optimal outcomes while minimizing the risks associated with open-heart surgery. They represent an important advancement in the management of complex CHD in adults, improving quality of life and potentially long-term prognosis.

## Pregnancy and family planning

In contemporary healthcare, most girls born with CHD will eventually reach their childbearing years (1, 37–39). However, for many women living with CHD, the prospect of pregnancy carries a degree of risk, both for the expectant mother and fetus (40–43). It becomes crucial that cardiovascular providers involved in the care of these women possess up-to-date knowledge in the management of women with CHD and pregnancy. This knowledge should encompass not only preconception counseling and diagnostic assessments to gauge maternal and fetal risk but also the ability to manage these patients effectively throughout their pregnancies. Equally important is their ability to recognize when it is prudent to refer these patients to regional centers specializing in pregnancy management for those with CHD.

There are significant changes in cardiovascular physiology that occur even in a normal pregnancy. The initial trimester is characterized by a notable reduction in peripheral vascular resistance, reaching its lowest point in the middle of the second trimester with a subsequent plateau or slight rise during the remainder of the pregnancy (44). As blood volume increases and systemic vascular resistance falls, cardiac output increases during this period. At the 24 week mark, cardiac output may potentially have increased by as much as 45% compared to baseline (45).

During these phases of pregnancy, women with CHD may face challenges in augmenting cardiac output appropriately to meet the demands of the fetus and their own bodies. They may also develop volume overload leading to heart failure (HF) exacerbations or develop arrythmias. Additionally, the completion of pregnancy does not necessarily result in the end of cardiovascular risk for the mother. The prolonged activation of the renin-angiotensin-aldosterone system and potential ventricular remodeling increases the risk of post-partum decompensation (46, 47). Additionally, the abrupt rise in vascular resistance post-delivery in the setting of increased plasma volume increases the risk of potential hemodynamic decompensation.

Not all pregnancies carry the same risk of cardiovascular compromise. Substantial work has gone into the area to determine the risk factors that place a woman at higher risk. The modified World Health Organization (WHO) classification has grouped patients into four pregnancy risk categories (Classes I-IV) based on their medical condition (48). Class I includes patients with no detectable increased risk of maternal mortality and either no or only a mild increase in morbidity, and they are not discussed further in this document. Class II comprises women who may experience a slight increase in maternal mortality or moderate morbidity with pregnancy. Class III individuals may face a significant rise in maternal mortality or severe morbidity, while those in Class IV could confront an exceptionally high risk of maternal mortality or severe morbidity, making pregnancy inadvisable. It is recommended that patients in Class IV receive counseling to avoid pregnancy, and if pregnancy is confirmed, termination should be considered.

The management of pregnancy, discussion of pregnancy contraception, and termination for pregnancies in the United States has gone and will continue to undergo extensive changes since the landmark Dobbs vs. Jackson Women's Health Organization decision (49). Following the decision, many states have swiftly moved to restrict abortion access, leading to a tumultuous legal landscape where the status of abortion rights is in constant flux (50).

Pregnancy counseling should continue to occur at nearly every visit in a woman of childbearing age. Choices in contraception both for routine and emergency contraception should be discussed and offered. For sexually active females our center's preference is consideration of long-acting reversible contraceptive (LARC) methods. LARC methods include the intrauterine device (IUD) and represent the most cost-effective reversible option to prevent unintended pregnancy (51). If oral contraception is preferred, prescriptions with no or low estrogen are preferred. The risks of hormonal preparations related to venous thrombus embolism (VTE) vary depending on the dose of estrogen, however the relative risk for thrombosis in patients who take oral contraceptive is three- to fivefold higher compared with that of nonuser (52).

Emergency contraception comes in several forms in the United States. The more well-known “Plan B” is levonorgestrel, a high dose progestin. This is particularly favorable given the lack of estrogen, does not carry the risk of thrombosis associated with estrogen containing pills, and is available over the counter. However emergency contraception pills may be less effective in overweight women. Copper-releasing IUDs, when implanted within 5 days of unprotected sex, are not only more effective than oral EC methods, but they also contribute significantly to reducing future unintended pregnancies and abortions (53).

It is also important to understand that not every pregnancy carries the same level of risk as outlined in the modified WHO consensus statement. Furthermore, the decision to move forward with pregnancy, contraception to avoid pregnancy, and decision to terminate or otherwise continue pregnancy in those with high cardiovascular risk is ultimately the choice of the patient. After providing the patient with comprehensive counseling that covers both maternal and fetal risks, if the decision to proceed with pregnancy is made, diligent monitoring and follow-up care should be conducted in collaboration with maternal fetal medicine, ACHD cardiologists, OB anesthesia, and any necessary specialists. Much like the heart team model for surgical or structural intervention, women with CHD in the setting of pregnancy often require a multi-disciplinary approach. Additionally, emotional, and psychological support is important as they may experience increase stress and anxiety during pregnancy which can also result in worsened maternal/fetal outcomes (54). Our own experience has shown that patients benefit from meeting with their ACHD physician at least each trimester and depending on the severity of their CHD benefit from imaging each trimester as part of medical management and delivery planning.

A Caesarean section (C-section) is not typically required in the delivery of patients with cardiac disease. Though, the mode of delivery should be carefully considered based on the individual patient's medical condition and the severity of the CHD. While there are situations where a C-section is medically necessary, vaginal delivery can be safe and appropriate for many women with CHD. A significant portion of the risk of maternal decompensation relates to the incidence of blood loss and volume shifts that can occur in delivery. Blood loss during a C-section tends to be higher than during a vaginal delivery. On average, blood loss during a planned or elective C-section can range from 500 ml–1,000 ml (approximately 17–34 ounces). In emergency C-sections or C-sections with complications, blood loss can be even greater, exceeding 1,000 ml (55) and obstetrical hemorrhage contributes significantly to maternal morbidity and mortality (56).

## ACHD and heart failure

ACHD patients are at an increased risk of developing HF over their lifetimes, particularly those with univentricular circulation (1). Excess mortality among adult patients with CHD due to HF, as compared with the general population is well described (57). Evidence indicates that signs of HF manifest in approximately 22% of adults who have undergone an atrial switch procedure (Senning or Mustard operation) for transposition of the great arteries (TGA), 32% of adults with congenitally corrected transposition (ccTGA), and 40% of adults who have undergone Fontan completion (58).

As such the diagnosis and treatment of HF is paramount in decreasing mortality and morbidity. As a first step, it is important to recognize that the initial congenital heart surgeries or palliative shunts may play a role in the development of HF in adulthood. As an example, surgical repair of tetralogy of Fallot often results in hemodynamically significant pulmonary regurgitation. This has been associated with right ventricular (RV) dilatation, biventricular dysfunction and arrhythmias (59). Patients with D-TGA s/p atrial switch have a systemic RV which places level of stress on the systemic ventricle for which it was not designed. And those with Fontan circulations have both the challenges of a univentricular system and the concomitant high rates of liver disease resulting in a myriad of complications. Given the varying nature of the disease processes dependent on the initial diagnosis and surgical palliations a one size fits all approach of traditional adult HF treatment of goal directed medical therapy is not possible or perhaps even reasonable.

In the initial evaluation of ACHD patients with HF one of the first keys to successful evaluation and treatment is addressing the need for potential transcatheter, surgical, or electrophysiologic intervention. In patients with RV dysfunction with large atrial septal defects, for example, should then undergo careful evaluation of closure of the defect. The diagnosis and treatment of a significant hemodynamic lesion is paramount in the treatment of HF in the ACHD population (58).When evaluating ACHD patients with suspected HF, in addition to assessment of a treatable hemodynamically significant structural abnormality, initial laboratory workup should be pursued. Notably, evaluation for iron deficiency should also be completed. Iron deficiency is present in approximately 50% of patients with symptomatic HF and is independently associated with worse functional capacity, lower quality of life and increased mortality (60). Additionally, iron deficiency anemia is common in ACHD patients and is associated with a 3-fold increased risk of death (61).

Individuals with impaired function of the systemic left ventricle often receive treatment in accordance with HF guidelines (62),which comprises a combination of ACE inhibitors or angiotensin II receptor blockers (ARBs), beta-blockers, mineralocorticoid receptor antagonists, SGLT-2 inhibitors, and, in some cases, sacubitril/valsartan as a replacement for ACE inhibitors or ARBs. While the precise effect of such therapeutic regimen is not well validated among patients with ACHD, it is conceivable that some benefit might be obtain due to shared pathophysiological pathways (63).

In individuals presenting with a systemic morphologic RV, the likelihood of HF progression over time is estimated to be present in 65% of these patients by the age of 45 years of age (64–66). Our institution adopts a cautious approach when considering the administration of conventional left HF medications to this specific patient group. However, it is worth noting that a combination of diuretics and SGLT-2 inhibitors can offer symptomatic relief. The utilization of beta blockers and ACE inhibitors is prevalent in this patient cohort, although there is limited available data supporting their effectiveness.

In patients with a functionally univentricular heart who have undergone palliation with a Fontan circulation, the presence of HF symptoms combined with impaired ventricular function necessitates treatment and symptom alleviation. Our approach remains consistent, regardless of whether the patient has a systemic morphologic left or right ventricle. The most significant benefits have been observed when targeting specific therapies to reduce pulmonary vascular resistance, such as phosphodiesterase inhibitors, or when lowering afterload with beta-blockers (58, 67). Diuretics are only recommended if there is evidence of fluid overload.

Additionally, it is important to recognize that Fontan associated HF is a distinct circulatory derangement with hemodynamic features that can mimic portal hypertension, but with the additional complication of limited ability to augment cardiac output (68). Protein losing enteropathy (PLE) may also develop. PLE is a condition where proteins leak into the intestines and are not properly absorbed. Fontan patients may develop PLE, which can lead to symptoms like diarrhea, edema, malnutrition, and further complications. Fontan-associated HF is a complex condition, and patients may experience a combination of these manifestations or additional complications. Early detection and intervention are crucial in the management of Fontan-associated HF to improve outcomes and quality of life for patients.

Finally, the criteria for selecting suitable ACHD patients for implantable cardioverter-defibrillator implantation as primary prevention for sudden cardiac death lacks precise definition, primarily because of limited data from randomized clinical trials. Consequently, current guidelines heavily depend on non-randomized studies and expert opinions to formulate their recommendations. Though certainly ACHD patients who survive sudden cardiac death or those with sustained ventricular arrythmia without an easily identifiable correctable cause would be considered as a strong indication for secondary prevention ICD placement.

In patients with normally related biventricular anatomy and a systemic left ventricle, the data for ICD implantation is much clearer given the large amount of data from adult cardiovascular trials. According to the PACES/HRS expert consensus statement, adults with congenital heart disease (CHD) and biventricular physiology, alongside a systemic left ventricular ejection fraction of ≤35% and New York Heart Association (NYHA) class II or class III symptoms, are indicated for an implantable cardioverter-defibrillator (ICD) (class I, Level of Evidence B, Table 1) (69). This recommendation is derived from the ACC/AHA guidelines regarding ICD usage in non-ischemic cardiomyopathy patients and should not be extended to individuals with single-ventricle physiology or systemic right ventricle (26).

**Table 1:** Recommendation for permanent pacing in adults with CHD as per PACES/HRS consensus (69).

Recommendations	
Class I	1. Permanent pacing is recommended for adults with CHD and symptomatic sinus node dysfunction, including documented sinus bradycardia or chronotropic incompetence that is intrinsic or secondary to required drug therapy (Level of evidence: C). Devices that minimize ventricular pacing are preferred (Level of evidence: B). 2. Permanent pacing is recommended in adults with CHD and symptomatic bradycardia in conjunction with any degree of AV block or with ventricular arrhythmias presumed to be due to AV block (Level of evidence: B). 3. Permanent pacing is recommended in adults with congenital complete AV block and a wide QRS escape rhythm, complex ventricular ectopy, or ventricular dysfunction (Level of evidence: B). 4. Permanent pacing is recommended for adults with CHD and postoperative high-grade second- or third-degree AV block that is not expected to resolve (Level of evidence: C)
Class IIa	1. Permanent pacing is reasonable for adults with CHD and impaired hemodynamics, as assessed by noninvasive or invasive means, due to sinus bradycardia or loss of AV synchrony (Level of evidence: C). 2. Permanent pacing is reasonable for adults with CHD and sinus or junctional bradycardia for the prevention of recurrent IART (Level of evidence: C). Devices with atrial antitachycardia pacing properties are preferred in this subpopulation of patients (Level of evidence: B). 3. Permanent pacing is reasonable in adults with congenital complete AV block and an average daytime resting heart rate <50 bpm (Level of evidence: B). 4. Permanent pacing is reasonable for adults with complex CHD and an awake resting heart rate (sinus or junctional) <40 bpm or ventricular pauses >3 s (Level of evidence: C). A device with antitachycardia pacing properties may be considered if the underlying anatomic substrate carries a high likelihood of developing IART (Level of evidence:B)
Class IIb	1. Permanent pacing may be reasonable in adults with CHD of moderate complexity and an awake resting heart rate (sinus or junctional) <40 bpm or ventricular pauses >3 s (Level of evidence: C). A device with antitachycardia pacing properties may be considered if the underlying anatomic substrate carries a high likelihood of developing IART (Level of evidence: B). 2. Permanent pacing may be considered in adults with CHD, a history of transient postoperative complete AV block, and residual bifascicular block (Level of evidence: C).
Class III	1. Pacing is not indicated in asymptomatic adults with CHD and bifascicular block with or without first-degree AV block in the absence of a history of transient complete AV block (Level of evidence: C). 2. Endocardial leads are generally avoided in adults with CHD and intracardiac shunts. Risk assessment regarding hemodynamic circumstances, concomitant anticoagulation, shunt closure prior to endocardial lead placement, or alternative approaches for lead access should be individualized (Level of evidence: B).

In patients with a systemic right ventricle or single ventricle physiology, the PACES/HRS guidelines offer a class 2b recommendation with a level of evidence based upon registry or observational studies, and certainly should not be considered standard of care. In adults with a single or systemic right ventricular ejection fraction <35%, considering an implantable cardioverter-defibrillator (ICD) may be deemed reasonable. This suggestion gains further support when additional risk factors, such as non-sustained ventricular arrhythmias, unexplained syncope, New York Heart Association (NYHA) function class II or class III symptoms, or a QRS duration ≥140 ms, are present. Though in our practice we do not offer routine ICD placement for patients with depressed single ventricle function or depressed systemic right ventricular function (69).

## Transplant

Due to the high incidence of HF among patients with ACHD, transplant may need to be considered as a therapeutic option. Unfortunately, ACHD patients being considered for transplant have numerous barriers. As a result, amongst all adult patients receiving cardiac transplantation only 3% are those identified as having CHD (70).

ACHD patients are at considerable disadvantage compared to patients with acquired cardiac defects when it comes to transplant listing (2). Patients with ACHD often require listing for heart transplant through “exception status” in the United Network for Organ Sharing (UNOS), particularly those with significantly deteriorating clinical status that have cardiovascular anatomy that is not suited for mechanical support (71–74). The current UNOS listing algorithm is designed to favor patients with traditional adult cardiomyopathy and is disadvantageous to CHD patients. Individuals with ACHD may face greater limitations when considering cardiac transplantation due to elevated antibody levels due to prior blood transfusions, which can restrict the availability of suitable donors or render transplantation unfeasible.

Moreover, due to the specific anatomy of these patients and the presence of multiple prior sternotomies, left assisted ventricular devices are rarely an option. Even when they could potentially be implanted with benefit, it should be carefully evaluated if adding an additional sternotomy surgery to the patient would be wise prior to heart transplantation.

Surgical risk for heart transplant is also increased in these patients. ACHD patients have higher than average peri-operative complication risk and mortality compared to traditional adult HF patients (75). Despite this, ACHD patients have similar, if not better long terms survival after transplant compared with patients with acquired cardiac defects (76, 77).

## Conclusions

The ACHD patient population grows every year and has surpassed the pediatric CHD population. They present unique challenges that demand specialized care and a multidisciplinary approach. The success of cardiac surgeries and catheter interventions in ACHD patients underscores the importance of tailored strategies and the expertise of a skilled healthcare team. Furthermore, the management of HF in ACHD individuals requires vigilant monitoring, early intervention, and a deep understanding of their specific cardiac anatomy. Cardiac transplantation, while a potential lifesaving option, is often hindered by the current UNOS listing algorithm, limited donor availability and antibody sensitization in ACHD patients. In light of these complexities, ongoing research, advancements in medical technology, and collaborative efforts among healthcare professionals remain crucial in improving the outcomes and quality of life for ACHD patients navigating these challenging medical scenarios.

Pregnancy can be a complex journey for women with CHD. A multidisciplinary approach involving cardiologists, obstetricians, and often maternal-fetal medicine specialists, is essential to assess the risks and tailor a personalized care plan. They also have specific considerations that should be addressed as related to options for birth control which require familiarity by the ACHD cardiologists in conjunction with their obstetric colleagues.
